# Polysaccharides from *Exocarpium Citri Grandis*: Graded Ethanol Precipitation, Structural Characterization, Inhibition of α-Glucosidase Activity, Anti-Oxidation, and Anti-Glycation Potentials

**DOI:** 10.3390/foods14050791

**Published:** 2025-02-25

**Authors:** Meizhen Chen, Juan Wang

**Affiliations:** School of Food Science and Engineering, South China University of Technology, Guangzhou 510641, China; meizhench@outlook.com

**Keywords:** *Citrus grandis* “*Tomentosa*”, polysaccharides, ethanol fractionation, structural analysis, α-glucosidase suppression, radical scavenging capacity, non-enzymatic glycosylation inhibition activity

## Abstract

The endocarp of *Exocarpium Citri Grandis* (ECG) is abundant in various bioactive components, such as polysaccharides; however, there are few studies on them. Thus, it is highly necessary to carry out further research on the structural characterization and biological activities of ECG polysaccharides (EPs), which are important bioactive substances. In this study, water-extracted EPs were precipitated by ethanol with final concentrations of 50%, 70%, and 90% (*v*/*v*), respectively. Three crude polysaccharides (EP50, EP70, and EP90) were fractioned successively. The three polysaccharide fractions were structurally elucidated and were investigated in vitro for their biological activities related to glucose metabolism containing inhibitory effects on α-glucosidase and non-enzymatic glycosylation and their antioxidant capacities. The main results are summarized as follows: (1) Gradient ethanol precipitation and physicochemical properties of EPs: The yields of EP50, EP70, and EP90 were 11.18%, 0.57%, and 0.18%, respectively. The total sugar contents were 40.01%, 52.61%, and 53.46%, and the uronic acid contents were 30.25%, 18.11%, and 8.17%, respectively. In addition, the three fractions had the same composition of monosaccharides, including rhamnose, arabinose, galactose, glucose, xylose, mannose, galacturonic acid, and glucuronic acid, with differences in the content of neutral and acidic monosaccharides. They all may be branched polymers and spherical conformation, and they were acidic polysaccharides containing esterified and non-esterified uronic acids, pyranose-form sugars, and glycosidic linkages of α-configuration and β-configuration, with esterification degrees of 32.25%, 28.82%, and 15.58%, respectively. Meanwhile, EP50, EP70, and EP90 were mainly amorphous, and the molecular conformation in solution was a spherical branching polymer without a triple helix structure. The EPs exhibited excellent thermal stability, with their structures remaining stable below 170 °C. (2) In terms of activity research, the results showed that EPs had a good α-glucosidase inhibitory effect with IC_50_ values of 1.17 mg/mL, 1.40 mg/mL, and 2.72 mg/mL, respectively, among which EP50 was the best. EP50, EP70, and EP90 displayed antioxidant activity by scavenging DPPH and ABTS radicals as well as oxygen radical absorbance capacity. Among them, EP90 had the strongest antioxidant activity. Furthermore, the EPs showed prominent effects on the inhibitory activity of non-enzymatic glycosylation. In summary, the research on the extraction of polysaccharide from ECG provides a technical reference for the further utilization of ECG resources. This study on antioxidant activity provides theoretical support for their use as a natural antioxidant. As oxidation and glycation are relevant to diabetic complications, the result of this work suggests that EPs may be effective in preventing and treating diabetic complications.

## 1. Introduction

*Exocarpium Citri Grandis* (ECG) is an immature or nearly mature dried exocarp of *Citrus grandis* “*Tomentosa*” or *Citrus grandis* (L.) *Osbeck*, a well-known Chinese herb recorded in Chinese Pharmacopoeia [1,2], which originates from Huazhou City in Guangdong province [3]. In ancient times, it garnered attention and application from the imperial court due to its unique properties and was titled one of the “Ten Major Guangdong Medicines” and “Southern Ginseng” [4]. In 2024, its official inclusion of ECG in the catalog of substances that serve both dietary and medicinal purposes signified the recognition of its dual attributes in both the food and pharmaceutical sectors. The establishment of its status in these two sectors further expanded the scale of the ECG industry in Huazhou City, with increasing emphasis on production, processing, research, and development. Meanwhile, the cultivated area of ECG is also continuously expanding, indicating a steady progression towards its establishment as a pillar industry in Huazhou.

**Figure 1:**
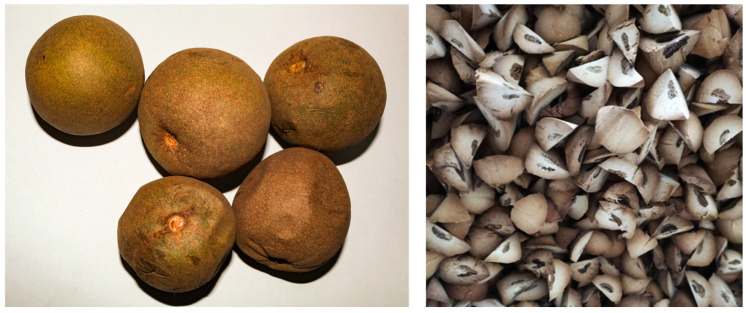
The whole fruits (**left**) and the endocarp cut into chunks (**right**) of *Exocarpium Citri Grandis*.

The traditional medicinal use of ECG involves utilizing its peel [2], while the endocarp portion is often unutilized during processing, resulting in a waste of resources; however, studies indicated that the endocarp of ECG also possesses various bioactive components, such as polysaccharides [5]. The chemical composition of ECG serves as the material basis for its pharmacological effects, necessitating further research into its functional components. As one of the important bioactive substances, ECG polysaccharides (EPs) have remarkable bioactivities such as antitussive, expectorant, anti-inflammatory, antioxidant, immunomodulatory, and antifatigue activities, which are closely correlated to chemical properties [6,7,8,9]. Therefore, it is very necessary to carry out further research on their structural characterization (Figure 1).

Polysaccharides are polar molecules that are generally soluble in water [10]. Thus, water is currently the most commonly used extraction solvent and serves as the basis for other extraction methods, such as acid-base extraction [11] and enzymatic hydrolysis [12]. The main principle of polysaccharide preparation through water extraction and ethanol precipitation involves disrupting the cell wall from the outside to the inside under mild conditions and extracting the polysaccharides from plants by exploiting the difference in solubility of polysaccharides under different polarity environments (such as water and ethanol) while simultaneously avoiding polysaccharide denaturation during the extraction process [13]. Recently, commonly used polysaccharide extraction methods include hot water extraction, acid-base extraction, enzymatic hydrolysis, ultrasound-assisted extraction, and microwave-assisted extraction, among which hot water extraction is the most frequently employed due to its ability to enhance the solubility of polysaccharides while causing minimal damage to them [14]. The method of single-step ethanol precipitation is mostly applied in the preparation of polysaccharides, which is not conducive to the subsequent separation and purification. In contrast, fractional ethanol precipitation facilitates the removal of impurities and reduces the loss of active ingredients. Additionally, many studies have found that ethanol concentration plays an important role in ethanol precipitation when preparing crude polysaccharides, which has been shown to have a significant impact on physicochemical properties and bioactivities [15]; therefore, polysaccharides of different factions can be separated by employing this method using various ethanol concentrations, which provide a more comprehensive revelation of the structural characteristics and biological activities.

In recent years, with the emphasis on physical health, there has been a growing interest in natural plant resources with bioactivities, in search of edible and medicinal resources that are devoid of adverse effects. Many plant polysaccharides exhibit inhibitory effects on non-enzymatic glycosylation reactions and possess antioxidant activities, such as *Ganoderma lucidum* polysaccharides [16], banana flower polysaccharides [17], pomegranate rind polysaccharides [18], and orange peel pectin polysaccharides [19]. As a natural plant resource that is both edible and medicinal, the study of ECG in relation to sugar metabolism-related activities has become an active research topic.

Nevertheless, to the best of our knowledge, there is no investigation that has thus far been done regarding the inhibitory effects on α-glucosidase and the non-enzymatic glycation of EPs, and their structural characteristics still remain unclear. Therefore, the present study aimed to extract three crude polysaccharides from the endocarp of ECG by graded ethanol precipitation, to structurally characterize them based on monosaccharide composition, molecular weight determination, infrared spectroscopy, nuclear magnetic resonance, and morphological properties, and to explore the inhibitory effect on α-glucosidase and non-enzymatic glycation and the antioxidant capacity, with an objective to accumulate relevant research data for EPs, which will promote the exploitation of this traditional herbal resource.

## 2. Materials and Methods

### 2.1. Materials and Reagents

Fruiting bodies of *Exocarpium Citri Grandis* (ECG) were harvested in February 2023, in the Modern Agricultural Industrial Park, Huazhou City, Guangdong Province, China. The harvested ECG was peeled, dried, and then pulverized using a Chinese herbal medicine high-speed pulverizer (FW-135, Tianjin Tester Instrument Co., Ltd, Tianjin, China). The ECG powder was degreased and decontaminated with petroleum ether and 95% ethanol and then dried for further use. 2,2-Diphenyl-1-trinitrohydrazine (DPPH), monosaccharide standards, 2,2′-azido-bis (3-ethylbenzothiazoline-6-sulphonic acid) diammonium salt (ABTS), ascorbic acid, macroporous resins, and gallic acid were supplied by Shanghai yuanye Bio-Technology Co., Ltd. (Shanghai, China). Shanghai Macklin Biochemical Technology Co., Ltd. (Shanghai, China) provided p-nitrophenyl-α-d-glucoside, glyoxal solution (8.8 M), sodium formate buffer, acarbose, α-glucosidase, and aminoguanidine hydrochloride. The rest of the chemicals used in this study were obtained from Sinopharm Chemical Reagent Co., Ltd. (Shanghai, China) and were of analytical quality.

### 2.2. Extraction of Polysaccharides by Graded Ethanol Precipitation

Following the method of Hou et al. [7], the crude polysaccharides were extracted from ECG by the traditional aqueous ethanol precipitation method. We weighed 20 g of defatted and decontaminated ECG exocarp, added 600 mL of distilled water, and oscillated it in a water bath at 100 °C for 2 h. After cooling to room temperature, centrifugation was performed at 3000 rpm for 10 min, and then it was pumped and filtered to obtain the ECG extract. We repeated all of the above once. Subsequently, the two extracts were combined and concentrated under reduced pressure to 1/9 of the original volume at 60 °C. Anhydrous ethanol was added under the stirring of a glass rod and stopped when the ethanol concentration reached 90% (*v*/*v*) in the mixture, which was allowed to be stored in a refrigerator at 4 °C for 12 h. After that, the extract was centrifuged at 3000 rpm for 10 min to collect filtrate, which was washed twice with anhydrous ethanol, acetone, and ether sequentially and dried in an oven at 50 °C, and we finally obtained the ECG crude polysaccharide extract.

The crude polysaccharide extract was prepared as 10 mg/mL solution, decolorized by macroporous resins, and concentrated to 1/6 of the original volume at 60 °C under reduced pressure, and a precipitate was obtained by adjusting the final ethanol concentration in the solution stepwise to 50%, 70%, and 90% (*v*/*v*). The three precipitates were redissolved in water and were freeze-dried after dialyzing for 72 h to obtain different ECG polysaccharides (EP) fractions named EP50, EP70, and EP90, respectively.

### 2.3. Physicochemical Properties

#### 2.3.1. Chemical Composition Analysis

The total sugar content of polysaccharides was determined by the phenol-sulfuric acid method [20]. The protein content was analyzed by Coomassie brilliant blue G-250 assay [21]. The content of uronic acid was evaluated by the *m*-hydroxybiphenyl method [22]. The Folin–Ciocaldeu method was used to determine the total polyphenol content [23]. The content of reducing sugar was determined by the DNS method [24].

#### 2.3.2. Esterification Degree Analysis

There were two methods, infrared spectroscopy [25] and chemical titration [26], employed to determine the degree of esterification of EPs. Considering that no relevant standard curve was established for determining the esterification degree using infrared spectroscopy, a secondary determination was conducted using the chemical titration method. The obtained esterification degree from the chemical titration was not significantly different from that measured by infrared spectroscopy, indicating a high degree of reliability in determining the esterification degree via infrared spectroscopy. The detailed experimental methods are provided in Supplementary Methods S1.

#### 2.3.3. Monosaccharide Composition Analysis

Monosaccharide composition was analyzed using high-performance anion-exchange chromatography (HPAEC) with an anion-exchange column (Dionex) and a pulsed amperometric detector (Dionex ICS 5000 system, Thermo Fisher Scientific, Waltham, MA, USA). Detailed experimental procedures are provided in Supplementary Methods S2.

#### 2.3.4. Measurement of SEC-MALLS-RI

The absolute molecular mass (M_w_), polydispersity coefficient (M_w_/M_n_), and radius of gyration (<S^2^>z^1/2^) of EPs were determined by using High-Performance Size Exclusion Chromatography-Multi-Angle Laser Light Scattering-Refractive Index Detection (HPSEC-MALLS-RI) coupled technique. Detailed experimental procedures are provided in the Supplementary Methods S3.

#### 2.3.5. Ultraviolet Spectral Analysis

Ultraviolet spectrophotometer (Agilent cary60, Santa Clara, CA, USA) was used to scan and analyze 0.5 mg/mL polysaccharide solution, with distilled water as blank control, and scanned in the wavelength range of 200–800 nm.

#### 2.3.6. Fourier Transform Infrared (FT-IR) Spectrometer

Fourier transform infrared (FT-IR) spectra of polysaccharides were collected using a spectrometer (Tensor 27, Bruker Optik GmbH, Leipzig, Germany) by mixing the polysaccharide powder with KBr powder for spectral analysis.

#### 2.3.7. Nuclear Magnetic Resonance (NMR) Spectroscopy Analysis

The sample was dissolved in 0.5 mL of D_2_O to obtain a 30 mg/mL polysaccharide solution. The 1D-NMR (^1^H NMR, ^13^C NMR) was recorded at 600 MHz using a Bruker AVANCE III HD 600 M spectrometer system (Germany).

#### 2.3.8. X-Ray Diffraction (XRD) Analysis

The physical properties of EPs were studied by multi-position automatic sampling X-ray diffractometer (PANalytical, Almelo, The Netherlands). The range of 2θ was 5–80°, and the scanning speed was 10°/min.

#### 2.3.9. Scanning Electronic Microscope (SEM) Observation

An appropriate amount of dried, unpowdered, freeze-dried original sample of EPs was taken with tweezers, and the sample was adhered to the table using a conductive adhesive, followed by gold plating, and observed in a vacuum environment by using scanning electron microscopy (Carl ZEISS EVO18, Oberkochen, Germany) with magnification of 50, 200, and 1000 times to observe morphologies of EPs.

#### 2.3.10. Atomic Force Microscopy (AFM) Observation

The microscopic morphology of EPs in aqueous solution was observed by atomic force microscope (Bruker Multimode 8). The sample was completely dissolved with distilled water, diluted to 1 μg/mL, and filtered through 0.22 μm filter membranes, and 4 μL of the filtrate was transferred to a freshly dissociated mica sheet and dried at room temperature overnight. Finally, the image processing software (NanoScope Analysis 1.8) was used for processing pictures.

#### 2.3.11. Chain Conformations

The chain conformation was determined by the SEC-MALLS-RI system and Congo red assay [27]. The specific experimental scheme of Congo red assay is in Supplementary Methods S4.

#### 2.3.12. Thermal Analysis

The thermogravimetric (TG) curve, derivative thermogravimetric (DTG) curve, and differential scanning calorimetry (DSC) curve of EPs were determined by a synchronous thermal analyzer (STA449F3, NETZSCH, Selb, Germany). The temperature was 25–800 °C, the heating rate was 10 °C/min, nitrogen was used as the protective gas, and the flow rate was 50 mL/min [28].

### 2.4. Bioactivities In Vitro

#### 2.4.1. Inhibition of α-Glucosidase Activity In Vitro

The inhibitory activity of EP on α-glucosidase was determined with the method by Chen et al. [29]. Supplementary Methods S5 lists the detailed experimental procedures.

#### 2.4.2. Antioxidative Capacity Assay In Vitro

The DPPH free radical scavenging activity was determined according to the method of Zhang et al. [30]. The ABTS radical scavenging activity of polysaccharides was determined according to the method reported by Shi et al. [31]. The oxygen-free radical absorption capacity of EPs was evaluated according to the method of Huang et al. [32]. The detailed experimental procedure is shown in Supplementary Methods S6.

#### 2.4.3. Anti-Glycation Assay In Vitro

Bovine serum albumin (BSA)-glucose (Glc) reaction system was used as a saccharification model to determine the inhibitory activity of EPs on non-enzymatic glycation [33]. Supplementary Methods S7 shows the detailed experimental procedure.

### 2.5. Statistical Analysis

Each group of experiments was tested three times in parallel, and the experimental results were expressed in the form of “mean ± standard deviation (SD)”. The data were plotted and analyzed using ORIGIN (2018, 64Bit) and IBM SPSS Statistics 27.

## 3. Results and Discussions

In addition to the monosaccharide composition analysis, all other measurement projects were conducted using intact polysaccharides without hydrolysis for research and analysis.

### 3.1. Yields and Compositional Characteristics of EPs

Ethanol precipitation is a commonly used method of polysaccharide extraction as a result of selectively precipitating polysaccharides based on their solubility characteristics. Polysaccharides with different chemical structures and compositions exhibit varying solubilities in water. For instance, polysaccharides with a higher proportion of hydrophilic groups (such as hydroxyl groups) have higher solubility in water and require the addition of more ethanol to precipitate out. Conversely, polysaccharides with a higher proportion of hydrophobic groups may exhibit the opposite trend. Therefore, by adjusting the ethanol concentration during the extraction process, polysaccharides of different compositions and properties can be selectively precipitated.

In this study, by adjusting the final ethanol concentration in the solution stepwise to 50%, 70%, and 90% (*v*/*v*), EPs, including EP50, EP70, and EP90, were obtained separately, and their extraction yields and chemical components are presented in Table 1. The yields of the EPs were ranked in descending order as EP50 > EP70 > EP90, depending on the different concentrations of ethanol, with notable differences ranging between 11.18% and 0.18%, indicating that EP50 was dominated. The total sugar content was highest in EP90 (53.46%), followed by EP70 (52.61%) and EP50 (40.01%). The three polysaccharides contained a small amount of protein that was less than 1.5%, suggesting that they might contain a few bound proteins. Meanwhile, EP90 contained the highest polyphenol (2.35%) compared to EP50 (0.46%) and EP70 (0.73%). Notably, all EPs consisted of uronic acid. In particular, EP50 had a significantly higher content of uronic acid (30.25%) than the others. They also contained some reducing sugar.

Additionally, the ζ potential of the EPs was negatively charged, indicating that they had an electron-donating effect. The higher the absolute value of the ζ potential, the higher the stability of the solution system [34]. When the absolute value of the ζ potential of all particles is greater than 30 mV, they will repel each other, and the dispersion is stable. In contrast, when the particle has a low ζ potential value (close to 0 mV), there will not be enough force to prevent particle aggregation [35]. The absolute values of the ζ potential of the three are all less than 30 mV, which means that their particles were unstable in the solution and tended to aggregate. The absolute value of the ζ potential of EP50 was relatively higher than those of EP70 and EP90, which might be due to its higher content of uronic acid. The results of this study are similar to the findings of Niu et al. [36], who employed a gradient ethanol method to isolate polysaccharides from *Mesona chinensis* and found that polysaccharide fractions with higher uronic acid content exhibited higher absolute ζ potential values, which may be attributed to the deprotonation of the carboxyl groups in uronic acids.

### 3.2. Monosaccharide Composition of EPs

**Figure 2:**
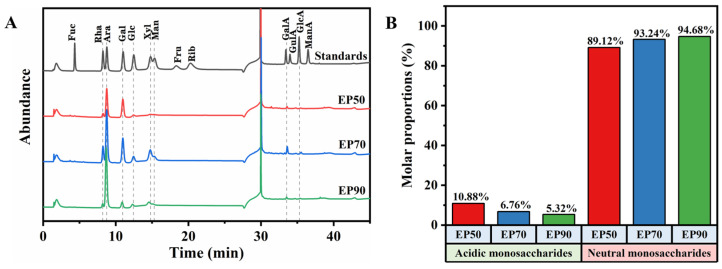
(**A**) HPAEC-PAD profiles of three EPs samples and monosaccharide standards. (**B**) The mole percentages of acidic monosaccharide and neutral monosaccharide of three EPs. Note: Neutral monosaccharides include Rha, Ara, Gal, Glc, Xyl, and Man. Acidic monosaccharides include GalA and GlcA.

As described in Figure 2A and Table 1, all the EPs were composed of eight kinds of monosaccharides, consisting of Rha, Ara, Gal, Glc, Xyl, Man, GalA, and GlcA, among which Ara and Gal were the dominant monosaccharides, of which the total molar ratios accounted for 51–73%. In terms of neutral monosaccharides, Ara and Gal were most abundant in the EPs, while the predominant acidic monosaccharides were GalA. The detected uronic acid suggested that they were acidic polysaccharides.

The low content of GalA (4.60–9.56%) of the EPs, which was different from some pectin polysaccharides fractionated from pericarps or endocarps of plants belonging to the same *Citrus* (Rutaceae), such as *Citrus Reticulata cv. Chachiensis* from which Peng et al. [37] extracted two polysaccharides using normal-temperature water and hot water with high GalA molar ratios of 55.26% and 55.7% and grapefruit, from which Wang et al. [38] extracted the pectin using hot water, which mainly consisted of GalA with a molar ratio of 68.36%.

As Figure 2B shows, EP50 has the lowest content of neutral monosaccharides, while both EP70 and EP90 exhibit higher contents of neutral monosaccharides. It was worth noting that the neutral monosaccharides increased, whereas the acidic monosaccharides of EPs declined with ethanol concentrations upward, which agreed with the findings of Guo et al. [39] who fractionated pectin from sugar beet. These results indicated that the polysaccharide fractions rich in neutral monosaccharides preferentially precipitated at relatively higher concentrations of ethanol, which may possess a greater capacity to enrich neutral polysaccharide fractions. In addition, polysaccharides rich in acidic monosaccharides may be less hydrated than those rich in neutral monosaccharides, such as EP50, and could be precipitated at lower ethanol concentrations, while neutral monosaccharide-rich polysaccharides needed to be extracted with higher concentrations of ethanol, such as EP90, which may be attributed to its stronger affinity for water molecules [40].

As evidenced, the method of graded ethanol precipitation did not alter the species of monosaccharides but caused a significant change in the proportions, which was consistent with the previous report [41].

D-xylose, D-glucose, D-galactose, L-arabinose, and D-mannose have been found by Cheng et al. [42] in the determination of the monosaccharide composition of ECG by adopting gas chromatography. However, the proportions of these monosaccharides in the present study differ significantly from their findings, which may be attributed to differences in raw material sources and testing methodologies.

### 3.3. Molecular Weight Distribution of EPs

**Figure 3:**
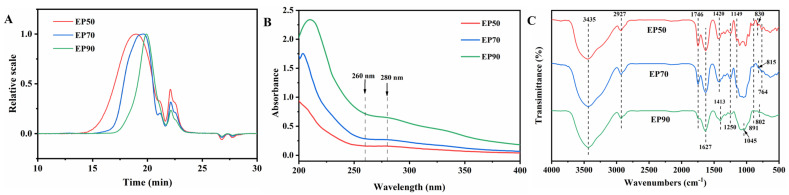
Elementary structure characterization of EPs. (**A**) HPSEC chromatograms; (**B**) UV spectra; (**C**) FT-IR spectra.

Figure 3A shows HPSEC chromatograms of the EPs. The molecular weight values, polydispersity index, and contents of all peaks are summarized in Table 1. The chromatogram of EP50 appeared with three peaks, corresponding to 50.83 kDa (accounting for 85.8%), 13.27 kDa (accounting for 4.7%), and 28.56 kDa (accounting for 9.5%), respectively, and EP70 also appeared with three peaks, corresponding to 40.53 kDa (accounting for 86.8%), 16.29 kDa (accounting for 3.8%), and 16.29% kDa (accounting for 9.3%), respectively, which implied that they mainly contained three components. Nevertheless, two peaks were observed for EP90, corresponding to 27.09 kDa (accounting for 89.5%) and 21.55 kDa (accounting for 10.5%), indicating that EP90 exhibited a higher purity than EP50 and EP70. It could be seen from Figure 3B that the broad peaks (Peak 1 in Table 1) were the highest molecular weight fractions, with a mass ratio of more than 85%, which were assumed to be the main components of EPs. Notably, the molecular weight (Peak 1) of EPs gradually decreased (EP50 > WP70 > EP90) with the increase in the concentration of ethanol. Chen et al. [43] prepared four *F. margarita* polysaccharide fractions, namely FP20, FP40, FP60, and FL80, through a fractional ethanol precipitation method with ethanol concentrations ranging from 20% to 80%. The molecular weights of these fractions were 1.341 × 10^6^ g/mol, 6.881 × 10^5^ g/mol, 6.000 × 10^5^ g/mol, and 1.941 × 10^5^ g/mol, respectively. Geng et al. [44] progressively adjusted the ethanol concentrations to 30%, 50%, and 80%, resulting in three lentinan polysaccharide fractions with molecular masses of 22,671 Da, 18,694 Da, and 6011 Da, respectively. The result of this study agrees with the findings of Chen et al. and Geng et al., indicating that high concentrations of ethanol may facilitate the precipitation of polysaccharides with lower molecular weights. This may be attributed to the fact that polysaccharides with higher molecular weight possess lower polarity, rendering them susceptible to precipitation by lower concentrations of ethanol, while polysaccharides with lower molecular weights exhibit better solubility, thus necessitating higher concentrations of ethanol for their precipitation.

On the other hand, as the concentration of ethanol was increased, the PDI of Peak 1 of the EPs was closer to 1, indicating that the molecular weight distributions were more and more concentrated, and the components of the EPs were more and more uniform. Based on the above-mentioned results, the concentration of ethanol significantly affected the molecular distribution of EPs.

### 3.4. The UV Spectra of EPs

Nucleic acids and proteins have maximum absorption peaks at 260 nm and 280 nm in the UV-vis spectrum [45]. The UV-vis spectrum of the EPs is shown in Figure 3B. It was observed that there was no absorption peak at 260 nm or 280 nm, indicating the presence of small amounts of proteins and the absence of nucleic acids in all three polysaccharide fractions, which was in accordance with the protein contents in Table 1.

### 3.5. FT-IR Spectra and DE Analysis

As illustrated in Figure 3C, three EP fractions had similar characteristics in the range of wavenumbers from 1250 cm^−1^ to 4000 cm^−1^. It could be seen that the three all had a major band at 3435 cm^−1^, which was attributed to the stretching of O-H, and a moderate band at 2927 cm^−1^ caused by stretching vibrations of C-H [46,47]. The absorption at around 1746 cm^−1^ and 1640 cm^−1^ could be clearly observed in all fractions, corresponding to the stretching vibration of C=O for esterified carboxyl groups (COOR) and ionic carboxyl groups (COO-), respectively, indicating the presence of uronic acid [48], which was consistent with the result of the monosaccharide composition of the EPs. The ratio of the absorption peak area at 1746 cm^−1^ to the total peak areas at these two absorption bands was often used to reflect the degree of esterification (DE) of polysaccharides [26]. As Table 1 shows, the DE of all three EP fractions was less than 50%, belonging to low-ester polysaccharides (<50%).

Moreover, it could also be found that there were little discrepancies in the FT-IR spectrum (Figure 3C) of the EPs, with wavenumbers ranging from 800 cm^−1^ to 1200 cm^−1^. The absorption peaks at about 1000–1200 cm^−1^ were related to the stretching vibrations of C-O-H, C-O-C, and C-C, which were characteristic of pyran ring structures [49]. The absorption peaks of three EP fractions in the “fingerprint” regions at 1149 cm^−1^ and 1045 cm^−1^ suggested that the presence of monosaccharides existed as pyranose forms in the EPs [30,50]. The amplitudes of the absorption peaks within the range of 1000–1150 cm^−1^ varied among the EPs, which may be attributed to the vibration of glycosidic bonds. The intensity of these peaks may reflect the degree of polymerization (the number of repeating units) within the polysaccharide chains. Specifically, EP50 exhibited two peaks in this region, while EP70 and EP90 displayed very weak peaks. This is presumably due to the lower molecular weights of EP70 and EP90 compared to EP50, suggesting that the polysaccharide chains of EP70 and EP90 may be shorter and have a lower degree of polymerization as a result of the weak stretching vibrations of EP70 and EP90 within the range of 1000–1150 cm^−1^. In addition, the bands at 891 cm^−1^ and 830 cm^−1^ were assigned to glycosidic bonds in the β- and α-configuration, respectively [51,52]. It could be demonstrated that EP50 (830 cm^−1^, 891 cm^−1^) and EP70 (815 cm^−1^, 891 cm^−1^) contained glycosidic bonds in the β- and α-configuration, while EP90 (802 cm^−1^) mainly contained glycosidic bonds in the α-configuration. From the above spectra assignments, three EP fractions extracted by graded ethanol precipitation exhibited similar characteristics, except for some characteristic bands that differed in adsorption intensities and wavenumbers, which may lead to some variances in the structures.

### 3.6. NMR Spectra of EPs

**Figure 4:**
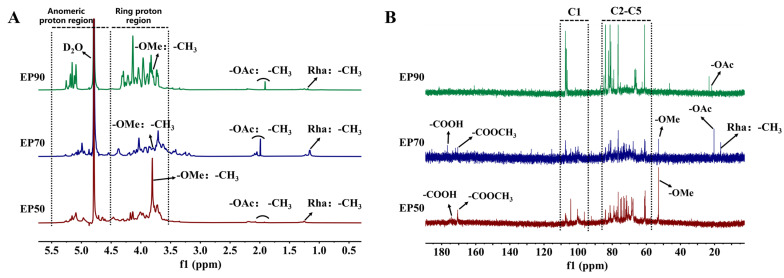
(**A**) ^1^H NMR spectrum; (**B**) ^13^C NMR spectrum.

The spectrum of ^1^H NMR (Figure 4A) and ^13^C NMR (Figure 4B) were used to further elucidate the structural characteristics of EPs. EP50, EP70, and EP90 all showed typical polysaccharide signals clustered in a narrow range of 3.5 to 5.5 ppm in ^1^H NMR and 60 to 105 ppm in ^13^C NMR [53].

In the spectrum (Figure 4A), signals between 5.5 and 4.5 ppm corresponded to the anomeric protons of the EPs, and these signals often served as signatures for differentiating complex carbohydrate structures [54]. The anomeric proton at δH 4.9–5.5 ppm indicated that the residue was in α-configuration, while the chemical shifts at δH 4.5–4.9 ppm were assigned to β-configuration [55,56]. The intensity of the signal peaks can reflect the content of residues. As shown in Figure 4A, the resonances of signals between δH 5.5 and 4.9 ppm were stronger than that in the region of the chemical shifts at δH 4.5–4.9 ppm, which corroborated that three EP fractions were primarily in α-anomeric configuration. A methoxy group (-OCH_3_) attached to the carboxyl terminus of GalA was established by the chemical shifts at δH 3.81 ppm, and two signals near δH 2.0 ppm were the acetyl signals attached to the O-2 and O-3 sites of GalA, respectively [57,58]. The signal near δH 1.20 ppm was characteristic of methyl linked to Rha, which was consistent with the monosaccharide composition of three EP fractions [59].

As depicted in ^13^C NMR (Figure 4B), signals occurring at approximately 175 ppm and 170 ppm of EP50 and EP70 indicated the presence of the carboxyl in acidic form and ester form, respectively, whereas no signals were found at these two chemical shifts of EP90, which might be attributed to the low content of uronic acid [60]. The chemical shifts occurring at the region of δC 95–110 ppm represented the anomeric carbons (C1), while the ring carbons were at δC 50–85 ppm [53]. One clustered peak was obviously found in the anomeric carbon regions (δC 95–110 ppm), corresponding to the presence of residues in furanose form in EPs [61]. Moreover, the signals at δC 107.3–109.3 ppm might be attributed to the C-1 of Ara(f) [56]. The spectra showed signals of the O-methyl (-OMe) of carboxylic acid methyl esters at near δC 52.81 ppm in the samples of EP50 and EP70 and the O-methyl (-OAc) of an acetyl group at δC 20.31 ppm in EP70 and EP90 [27,62]. The signal of the C6-methyl of Rhap at δC 16.38 ppm was also found in EP70 [63].

Overall, the stronger signals of ^1^H NMR and ^13^C NMR in EP90 were indicative of its higher purity compared to EP50 and EP70, confirming that graded alcohol precipitation was an effective way for the preliminary isolation and purification of polysaccharides. The intensity of the carbon spectra of EP70 was weak, which might be caused by its poor solubility. The stronger signals of the methoxy group (-OMe) in EP50 were due to its higher degree of methyl esterification. However, ^13^C NMR of EP50 and EP90 did not completely correspond to ^1^H NMR as a result of the influence of the impurities of the crude polysaccharides and the nuclear magnetic test conditions. The structural features of the three EPs remain to be further investigated.

### 3.7. Morphological Observations of EPs (SEM, AFM)

#### 3.7.1. SEM Analysis

**Figure 5:**
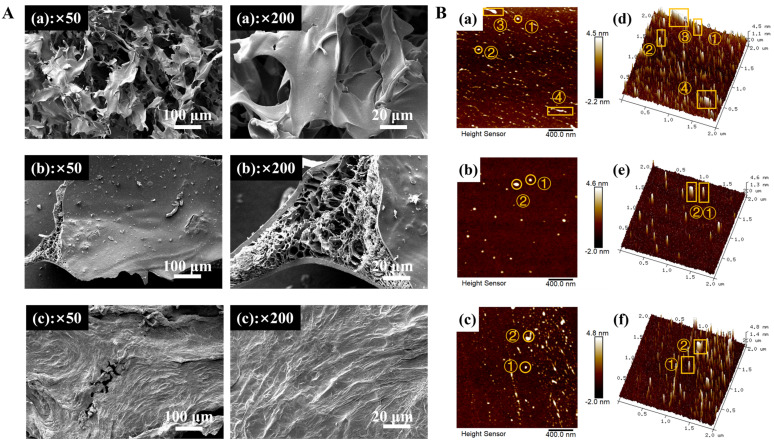
Morphological observations of EPs. (**A**) Scanning electron micrographs images of EP50 (**a**), EP70 (**b**), and EP90 (**c**) at different magnifications (×50 and ×200); (**B**) Atomic force microscopy images of EP50 (**a**,**d**), EP70 (**b**,**e**), and EP90 (**c**,**f**).

The surface morphologies of the EPs were observed by SEM at 50- and 200-times magnification. As shown in Figure 5A, the EPs were easy to curl and formed various irregular shapes after freezing, and significant discrepancies in shape and size were presented. EP50 had a lamellar structure bonded to each other. EP70 exhibited a massive structure with many internal cavities. EP90 had a large, flaky, morphological structure with a rough surface and cracks. The obvious differences between the three EP fractions might be related to molecular weight [36], and polysaccharides with larger molecular weights had an easier time establishing the hydrogen bonds between molecules with a relatively aggregated state, resulting in a flaky structure, such as in EP50 [64]. With the increase in the concentration of ethanol, some internal holes and cracks appeared in the structure of EP70 and EP90 as a consequence of the decrease in the molecular weight of EPs, leading to the diminution of the interaction force and molecular crosslinking degree, which was in agreement with the findings of Han et al. [65], who extracted polysaccharides from bamboo fungus using graded ethanol precipitation.

#### 3.7.2. AFM Analysis

AFM was used to observe the microscopic surface morphology of the EPs. As can be seen from Figure 5B, all EP fractions had granular or spherical particles and aggregates with irregular shape and size (①, ②), a stable structure that might be ascribed to the orientation to rotate and curl of polysaccharide molecules in the solution [66]. The spherical conformation of the polysaccharides suggests a potential branched structure [67]. The spherical structures observed in the 2D AFM images (a, b, and c: ①) of EP50, EP70, and EP90 indicated that all three possessed a branched structure, with branching points present. In particular, EP50 appeared to have a short-chain structure, and the reason might be that it had more uronic acid than EP70 and EP90, which could form intermolecular hydrogen bonds between the strong electronegative oxygen atom on the carboxyl of the uronic acid and the hydroxy hydrogen on the other chain of polysaccharides, which was similar to the report of Wang et al. [40], who extracted polysaccharides from soy hull by graded ethanol precipitation.

Additionally, the particle size range, height range, and average roughness of the EPs were summarized in Table 2. The heights of the EPs were far higher than the height of a single chain of polysaccharides (0.1–1 nm) according to the literature [52], indicating that the EPs might be branched and entangled with each other. As the concentration of ethanol increased, the maximum height and average roughness of the EPs decreased, which might correspond to the reduction of molecular weight (EP50 > EP70 > EP90) agreeing with Yao et al. [68].

Based on these results, it could be deduced that the extraction method of graded ethanol precipitation had a remarkable effect on the height, average roughness, and aggregability of polysaccharide molecules of the EPs.

### 3.8. Chain Conformation of EPs (SEC-MALLS-RI System and Congo Red Test)

#### 3.8.1. SEC-MALLS-RI System

**Figure 6:**
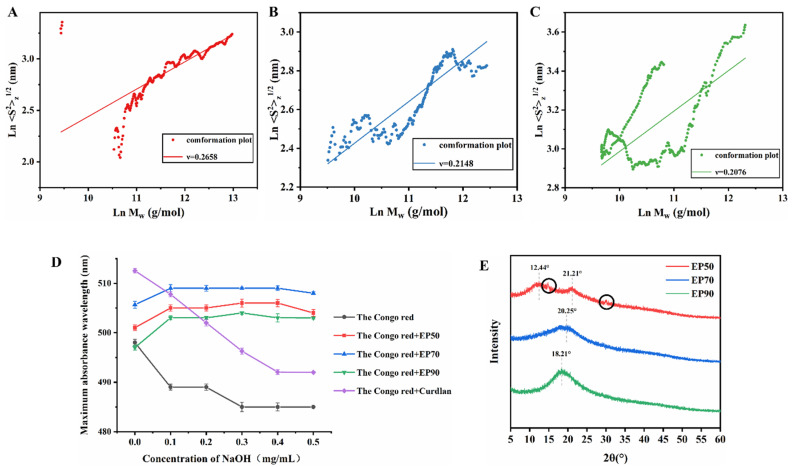
Advanced structure characterization of EPs. Double logarithmic plot of molecular weight against radius of gyration of EP50 (**A**), EP70 (**B**), and EP90 (**C**); (**D**) Maximum absorption wavelength of Congo red-EP complex at various concentrations of NaOH solution; (**E**) X-ray diffraction diffractogram.

Based on the theory of dilute polymer solutions, the chain conformation of polysaccharides could be analyzed. The values of the absolute molecular mass (M_w_) and the radius of gyration (<S^2^>z^1/2^) in dilute polymer solution were determined by the SEC-MALLS-RI system, from which the power law function <S^2^>z^1/2^ = kM_w_ν could be estimated. Figure 6A–C illustrates the double logarithmic plot of <S^2^>z^1/2^ against M_w_ of EP50, EP70, and EP90, respectively. The exponent values of ν were calculated and are displayed in Table 3 and related to the shape and conformation of the macromolecules in the solvent [69]. In terms of chain conformation, the ν values of 0.2–0.4, 0.3, 0.5–0.6, and 0.6–1.0 usually reflect the chain conformation for branching polymers, spherelike, flexible polymers, and semi-flexible chains, respectively [70,71,72], while the exponents of 0.33, 0.50–0.60, and 1.0 reflect those for the polymer molecular shapes of spheres, random coils, and rigid rods, respectively [73]. In view of the data (Table 3), all EP fractions existed as a globular shape with branches (ν < 0.3), which corresponds to the AFM analysis presented in Section 3.7.2. Furthermore, EP90 possessed the smallest exponent (ν), indicating that the degree of branching of EP90 may be higher than that of EP50 and EP70. As Figure 6C shows, the experimental points exhibit a “U”-shaped curve, which has been repeatedly reported in highly branched polymers [74], further confirming that EP90 is a highly branched polymer, similar in conformation to the polysaccharide from *Ganoderma lucidum* prepared by Hu et al. [70].

#### 3.8.2. Congo Red Assay

Congo red assay has been widely used to determine the helical conformation of polysaccharides [75]. It can react with polysaccharides that have a triple-helix conformation to form a complex in a certain range of NaOH concentration whose maximum absorption wavelength of this mixture system increases compared with the single Congo red solution [76], and then, with the increase in the NaOH concentration, the maximum absorption wavelength of the system decreased since the hydrogen bonds between the polysaccharide were disrupted, causing the triple-helix conformation of the polysaccharide to gradually disintegrate into a single coiled conformation [77]. Therefore, the chain conformation of polysaccharides in aqueous solution can be characterized by the change in the wavelength of maximum absorption in the Congo red assay.

As Figure 6D shows, Curdlan was used as a positive control in this experiment with a typical β-trihelix structure [78]. When the concentration of NaOH in the system was 0 mol/L, EP90 exhibited no significant increase in its maximum absorption wavelength compared to the blank control, and its maximum absorption wavelength remained unchanged with increasing NaOH concentrations, suggesting that EP90 did not possess a triple-helix structure. EP50 and EP70 displayed similar trends in their maximum absorption wavelengths: when the NaOH concentration is 0 mol/L, the maximum absorption wavelengths of both were higher than that of the blank control group, which may be due to the weak acidity of the aqueous solutions of EP50 and EP70, as the maximum absorption wavelength of Congo red increases with decreasing pH [79]. As the concentration of NaOH increased, the maximum absorption wavelengths of EP50 and EP70 remained unchanged, indicating that neither EP50 nor EP70 possessed a triple-helix structure. This finding is consistent with literature reports stating that polysaccharides with a molecular weight of less than 90 kDa typically do not exhibit a triple-helix structure [76].

### 3.9. X-Ray Diffractometry (XRD) Analysis of EPs

As presented in Figure 6E, two broad peaks at 12.44° and 21.21° with two small sharp narrow peaks at 14.75°and 29.93° (as shown by the diffraction peaks circled in Figure 6E) indicated that EP50 might contain a trace crystal structure. A weak peak at 20.25° of EP70 and a single peak at 18.21° of EP90 with different intensities were also observed, which was similar to the findings of Fu et al. [80], who extracted polysaccharides from blue honeysuckle berries (18.9°). The diffraction peaks and the intensities of EP50, EP70, and EP90 were different, which might be associated with different component contents in the polysaccharides changed by graded ethanol precipitation. However, since the peak intensities of the three were generally low, and the peak shapes were wide, it was conjectured that the EPs were predominantly amorphous with little microcrystalline structures.

### 3.10. Thermodynamic Properties of EPs

**Figure 7:**
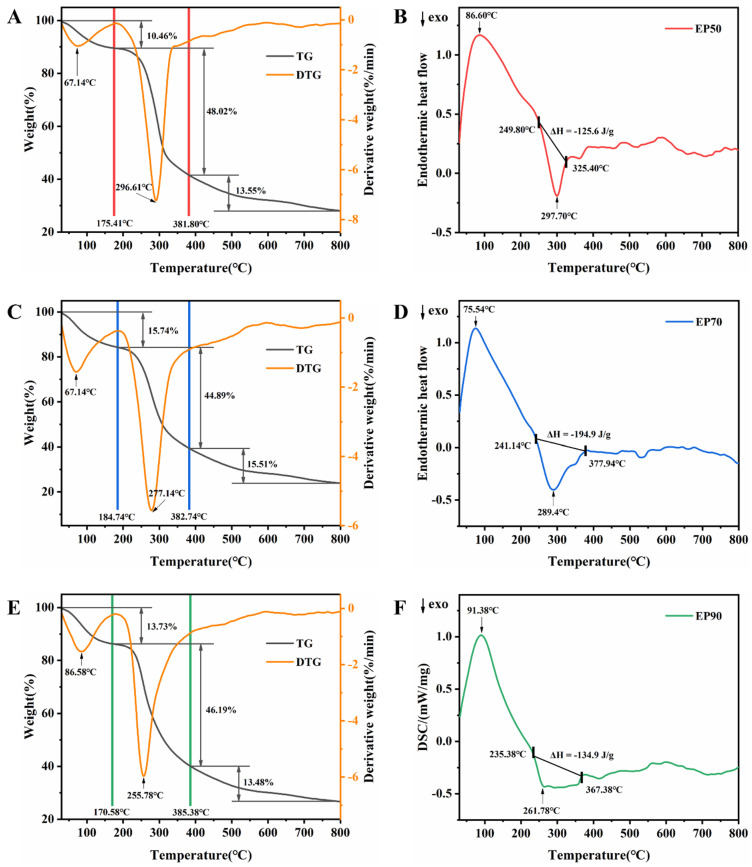
Thermogravimetric analysis curves and differential scanning calorimetry curves of EP50 (**A**,**B**), EP70 (**C**,**D**), and EP90 (**E,F**).

The thermogravimetry (TG), derivative thermogravimetry (DTG), and differential scanning calorimetry (DSC) curves of the EPs are shown in Figure 7.

As Figure 7A,C,E show, the EPs experienced three main stages of mass loss, with the first stage occurring at 30–180 °C, which was attributed to the loss of free water, bound water, and other volatile compounds of the EPs and the second rapid mass loss stage occurring at 170–385 °C, which was related to the thermal decomposition of the polysaccharides [81,82]. When the temperature was higher than 385 °C, the mass loss rate of the EPs (EP50, EP70, and EP90) gradually slowed down and finally tended to be stable when the final residual masses were 27.97%, 23.86%, and 26.60% (*w*/*w*), respectively. Long et al. [83] postulated that under a nitrogen atmosphere, the final residual should consist primarily of carbon, with its mass corresponding to the content of neutral monosaccharides. However, in the present study, the order of final residual masses among the three samples (EP50 > EP90 > EP70) did not align with the order of the neutral monosaccharide contents (EP90 > EP70 > EP50) referred to in 3.2. This discrepancy may be attributed to the presence of high-temperature-resistant impurities and a small amount of ash within the polysaccharides. As presented in the DTG curves, the maximum mass loss rates in the second stage appeared at 296.61 °C, 277.14 °C, and 255.78 °C of EP50, EP70, and EP90, respectively, which were presented thermal decomposition temperatures, demonstrating that the thermal stability of the EPs was in the descending order of EP50 > EP70 > EP90. The EPs, especially EP50, possessed more preferable thermal stability than commonly used commercial κ-carrageen (250 °C) [84], which grants the EPs broader potential for use in various fields such as food processing, pharmaceutical formulations, and biomaterials.

As Figure 7B,D,F show, the EPs exhibited an endothermic behavior at 30–250 °C generally related to the evaporation of water and an exothermic behavior at 250–380 °C associated with the depolymerization and self-degradation of the EPs’ structures, corresponding with the weight loss of polysaccharides in the TG curve.

Chen et al. [85] found that the bonds between uronic acids in polysaccharides are more stable and heat-resistant than those between neutral monosaccharides; therefore, polysaccharides with higher uronic acid content exhibit better thermal stability, which may be the reason that EP50 exhibited the strongest thermal stability. Meanwhile, there is also the notion that polysaccharides with larger molecular weights are more stable [86]. EP50 has the largest molecular weight among the three, which may also be one of the reasons for its strong thermal stability.

From the above results, the structures of the EPs remain thermally stable below 170 °C, which can meet the requirements of most food industrial applications.

### 3.11. Bioactivities In Vitro of EPs

#### 3.11.1. Inhibitory Effect on α-Glucosidase

**Figure 8:**
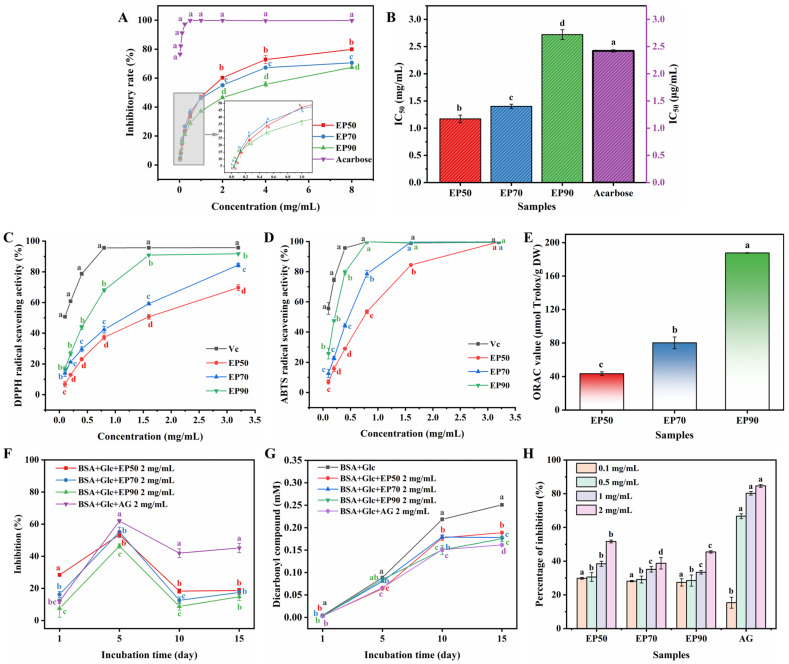
Biological activities of EPs. Inhibitory rates (**A**) and IC_50_ values (**B**) of EPs and acarbose on α-glucosidase; DPPH (**C**) radical and ABTS (**D**) radical scavenging activity of EPs and ascorbic acid (VC); (**E**) The oxygen radical absorbance capacity (ORAC) of EPs; Inhibitory effect of EPs and aminoguanidine (AG) on the formation of Amadori product (**F**), dicarbonyl compounds (**G**) and AGEs (**H**). Note: (1) The right axis indicates the IC_50_ value of acarbose in (**B**). (2) In (**A**,**C**,**D**,**F**,**G**) different lowercase letters indicate the existence of significant differences (*p* < 0.05) between data. (3) In (**H**), the different lowercase letters on the same colored bar chart indicate statistically significant differences between experimental groups with the same concentration (*p* < 0.05).

As depicted in Figure 8A, the inhibition effect of the EPs on α-glucosidase was concentration-dependent. The inhibition ratios of the EPs (EP50, EP70, and EP90) at 8 mg/mL on α-glucosidase were (79.87 ± 0.42)%, (70.55 ± 0.51)%, and (67.38 ± 0.22)%, respectively, in which EP50 exhibited the strongest inhibitory effects with the lowest IC_50_ value of 1.17 mg/mL, followed by EP70 (IC_50_ = 1.40 mg/mL) and EP90 (IC_50_ = 2.72 mg/mL), showing a downward order of EP50 > EP70 > EP90 on the activities against α-glucosidase as presented in Figure 8B. Although all EP fractions had lower inhibitory effects than acarbose, in contrast to polysaccharide fractions from *Chaenomeles speciosa* seeds (IC_50_ = 4.59 mg/mL) and *Fructus Mori* (IC_50_ = 11.31 mg/mL) [87,88], they showed a higher capacity against α-glucosidase due to their lower IC_50_ values, indicating that EPs could be a natural source of α-glucosidase inhibitor in the future.

The highest α-glucosidase inhibitory activity of EP50 may be attributed to its higher galacturonic acid content, potentially due to the carbonyl group of uronic acids facilitating the binding of polysaccharides to the enzyme [89]. Additionally, the differences in α-glucosidase inhibitory abilities of EPs may be correlated with their molecular weights. Xiong et al. [90] prepared *Lycium barbarum* polysaccharides using different solvents and found that polysaccharide fractions with larger molecular weights exhibited stronger α-glucosidase inhibitory activities. In this study, among the three crude polysaccharides from ECG, EP50 possessed the largest molecular weight and the strongest α-glucosidase inhibitory activity, aligning with the observations of Xiong et al. The aforementioned findings indicate that there exist differences in the α-glucosidase inhibitory activities among EP50, EP70, and EP90, which may be linked to their monosaccharide compositions and molecular weights [88,91].

#### 3.11.2. Antioxidant Activities of EPs

To estimate the effect of different concentrations of ethanol on the antioxidant activities of EPs, DPPH, and ABTS radical scavenging ratio and oxygen radical absorbance capacity of EPs were determined and compared and are illustrated in Figure 8C, D, and E, respectively. In this study, the scavenging effects of EPs on DPPH and ABTS radicals were measured with ascorbic acid (VC) as a positive control.

As noted in Figure 8C, the scavenging activity of EPs and VC on DPPH radicals was related to their concentration and their scavenging activity increased with the increasing concentration within 0.1–1.6 mg/mL. At the maximum test concentration of 3.2 mg/mL, the scavenging effect of EP50, EP70, and EP90 increased to 69.64%, 84.40%, and 91.75%, respectively, in which EP90 exhibited the highest scavenging ratio, indicating that it had a much better scavenging DPPH radical capacity than that of other polysaccharides.

As Figure 8D presented, the ABTS radical scavenging activity of the EPs was evaluated. It was observed that three EP fractions displayed significant scavenging effects on ABTS radicals at all tested concentrations in a dose-dependent manner. The scavenging activity of VC was stronger than that of all EP fractions, with its clearance approaching 100% at a low concentration. Moreover, among the three EP fractions, EP90 possessed the strongest ABTS scavenging capacity and at the concentration of 0.8 mg/mL, the clearance of EP90 reached 99.87%, which was comparable to VC (100%). The above results implied that EPs might act as an electron or hydrogen donator to scavenge DPPH and ABTS radicals.

The magnitude of the ORAC value reflects the antioxidant capacity of the measured substance, and the greater the value, the greater the antioxidant capacity [92]. As Figure 8E shows, EP90 possessed the strongest capacity for oxygen radical absorbance with the highest ORAC values of 187.67 μmol Trolox/g DW, followed by EP70 (80.18 μmol Trolox/g DW) and EP50 (43.56 μmol Trolox/g DW). Compared with other plant polysaccharides, such as sulfated polysaccharides from seaweeds (2–10 μmol Trolox/g DW), water-soluble polysaccharides from the leaves and stems of *Rabdosia serra* (7~40 μmol Trolox/g DW), and polysaccharides from *Laminaria japonica* (123.14 μmol Trolox/g DW) [93,94,95], it is speculated that the oxygen free radical absorption capacity of EP50 was of the medium level, while those of EP70 and EP90 were of a higher level.

Among the three EP fractions, EP90 exhibited the strongest antioxidant activity which may be attributed to its lower molecular weight, possessing better water solubility and more reductive hydroxyl group terminals to accept and eliminate radicals [96,97]. On the other hand, it has been reported that the antioxidant activity of neutral monosaccharides is stronger than that of acidic monosaccharides [98], which might also be one of the reasons for that. EP90, possessing multiple antioxidant-promoting factors, exhibits broad application prospects in fields such as food preservation, pharmaceutical, and healthcare products, as well as cosmetics. Particularly in scenarios requiring efficient scavenging of free radicals, the application potential of EP90 stands out prominently.

#### 3.11.3. Inhibitory Effect of EPs on Non-Enzymatic Glycation in BSA-Glucose System

The BSA-Glucose system was used to estimate the inhibitory effect of EPs on non-enzymatic glycation in vitro, measured with aminoguanidine (AG) as the positive control. In general, non-enzymatic glycation includes three stages: early, intermediate, and end stages corresponding to three kinds of products, which were Amadori products, dicarbonyl compounds, and advanced glycation end products (AGEs), respectively [99]. The representative products were detected to evaluate the inhibitory ability of anti-glycation at every stage.

As presented in Figure 8F, the EPs and AG exhibited inhibitory effects on the formation of Amadori products. All samples displayed an increasing trend of inhibitory percentage, with the incubation time proceeding from the first to the fifth day and then decreasing to different levels on the tenth day, which might be caused by the depolymerization of polysaccharides by free radicals produced in the non-enzymatic glycation [100]. Overall, the inhibitory effect on the formation of Amadori products was indicated to be in the order of AG > EP50 > EP70 > EP90 throughout the period of incubation when all samples were at the same concentration (2 mg/mL). The maximum inhibition rates of EPs (EP50, EP70, and EP90) on Amadori products were found on the fifth day of the incubation time and were 53.47%, 55.12%, and 46.30%, respectively.

In the intermediate stage, dicarbonyl compounds were generated by the Amadori products through oxidation and dehydration reactions, which were active and easily continued to produce AGEs [101]. As Figure 8G shows, the content of dicarbonyl compounds in EPs and AG gradually increased during the 15 days of incubation. It was observed that from the 10th to the 15th day of the incubation time, the blank group (BSA + Glc) still maintained an upward state, while the intervention group (containing EPs or AG) tended to be stable and no longer grew, indicating that EPs and AG exhibited the inhibitory effect on dicarbonyl compounds. On the 15th day of the culture, AG showed the lowest level of dicarbonyl compounds, which was 0.1615 mmol/L, followed by EP90 (0.1751 mmol/L), EP70 (0.1781 mmol/L), and EP50 (0.1888 mmol/L).

In the late stage, AGEs were irreversibly generated, resulting from the reaction occurring between the dicarbonyl compound and the free amino groups of proteins [102]. As it was depicted in Figure 8H the inhibition percentages of EPs and AG on AGEs were determined after 15 days of culture. EPs and AG exhibited stronger inhibitory capacity on the formation of AGEs within the increase of test concentrations, which was dose-dependent. When the test concentrations of AG and EPs were at 2 mg/mL, the inhibitory ratios of EP50 (51.6%), EP70 (38.74%), and EP90 (45.47%) on AGEs were weaker than AG (84.63%). However, the inhibitory rates of EPs (EP50, EP70, and EP90) on AGEs were 38.45%, 35.15%, and 33.41%, respectively, which was close to the polysaccharides extracted from *Dendrobium huoshanense* (35%) at the same dose (1 mg/mL) [103].

Furthermore, studies have reported that the excessive production of free radicals will accelerate the non-enzymatic glycation resulting in the accumulation of AGEs in bodies [104]. Based on that, it has been speculated that the activity of inhibiting non-enzymatic glycation was related to the antioxidant capacity [105]. In this investigation, the orders of antioxidant ability were not the same as those of anti-glycated effects in all three stages of AGE formation, which was different from the findings of Zhu et al. [106] and Zhang et al. [88]. It was deduced that the antioxidative ability might not be the unique mechanism of the inhibitory effect on the non-enzymatic glycation of EPs. Currently, there are AGE inhibitors available in the market that exhibit stronger inhibitory capabilities than EPs; however, they possess certain side effects on the human body. Consequently, there is a growing interest in biologically active natural plant products in search of edible and medicinal resources that are devoid of adverse reactions. EPs, polysaccharides extracted from ECG that are both edible and medicinal, possess the ability to inhibit non-enzymatic glycosylation, and these two characteristics offer potential possibilities for the development of novel drugs targeting diabetes and aging.

## 4. Conclusions

In this study, three EPs were prepared using a gradient ethanol precipitation method, and a preliminary investigation into their structural characteristics was conducted. The results revealed that these three crude polysaccharides are predominantly composed of neutral monosaccharides. As the concentration of ethanol increased, the EPs exhibited trends of increasing neutral monosaccharide content, decreasing uronic acid content, decreasing degree of esterification, and reduced molecular weight. Their molecular conformations are likely to be spherical branched polymers without a triple-helical structure. These findings fill a gap in the structural research of crude polysaccharides from ECG.

EPs exhibit excellent thermal stability, with their structures remaining stable below 170 °C, implying that in the field of food processing, they can withstand higher temperatures without undergoing structural changes, which is beneficial for maintaining the taste and nutritional value of foods. In pharmaceutical formulations, they can serve as stabilizers or excipients to enhance the stability and bioavailability of drugs. In the realm of biomaterials, the thermal stability of EPs offers the possibility of preparing stable biomaterials under high-temperature conditions.

In this study, the bioactivities related to the glucose metabolism of EP50, EP70, and EP90 were different. Specifically, EP50 exhibited good inhibitory activity of α-glucosidase, while EP90 demonstrated strong antioxidant activity. All EPs exhibited inhibitory effects on the three stages of non-enzymatic glycosylation and their mechanisms of inhibition may not be limited to antioxidation alone. This research provides important insights into the applications of EPs. In the food industry, EP50’s notable ability against α-glucosidase can be utilized for the development of functional foods and dietary supplements. Meanwhile, EP90’s antioxidant activity and inhibitory effect on non-enzymatic glycosylation make it a potential candidate for use in cosmetics, where it can reduce free radical damage and delay skin aging.

However, this study also has several limitations:

(1) It can be seen that the structural characteristics of the extract are determined by the solvent. The concentration of ethanol plays a crucial role in the extraction process of EPs. Future studies will further subdivide the concentration of ethanol to explore the differences in structural characteristics and biological activities of EPs extracted at various ethanol concentrations.
(2) Structure determines function. The difference in structures of EP50, EP70, and EP90 may lead to discrepancies in their bioactivities. However, the current interpretation of the structure-activity relationship of polysaccharides from ECG is not yet thorough. Existing studies typically speculate on the relationship between structural differences and activity, but whether there is a deeper connection between them still requires investigation through technical means such as molecular docking and molecular modeling.
(3) There is limited research on the activities of polysaccharides from ECG. The elaboration on the biological activities of EPs in this study is based on in vitro and chemical experiments. The mechanisms of these bioactivities require support from more mechanistic experimental data.

## Figures and Tables

**Table 1:** Yield, chemical composition, and molecular weight of EPs by graded ethanol precipitation.

Samples	EP50	EP70	EP90
Raw material (ECG)	** 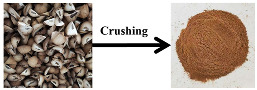 **		
Optical photos (EP)	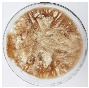	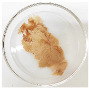	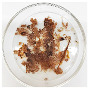
Yield (%) ^A^	11.18 ± 0.57 ^a^	0.57 ± 0.26 ^b^	0.18 ± 0.03 ^b^
Chemical composition			
Total sugar (%) ^A^	40.01 ± 2.28 ^b^	52.61 ± 0.67 ^a^	53.46 ± 0.96 ^a^
Uronic acid (%) ^A^	30.25 ± 1.52 ^a^	18.11 ± 1.83 ^b^	8.17 ± 1.82 ^c^
Protein (%) ^A^	0.46 ± 0.03 ^b^	0.25 ± 0.09 ^b^	1.05 ± 0.16 ^a^
Reducing sugar (%) ^A^	9.37 ± 0.69 ^a^	10.16 ± 0.39 ^a^	9.35 ± 0.35 ^a^
Polyphenol (%) ^A^	0.46 ± 0.02 ^b^	0.73 ± 0.09 ^b^	2.35 ± 0.18 ^a^
Zeta potential (mV) ^A^	−28.20 ± 4.41 ^a^	−24.00 ± 3.26 ^a^	−19.80 ± 3.55 ^a^
DE (%) ^A, B^	32.25 ± 0.16 ^a^	28.82 ± 0.52 ^b^	15.58 ± 0.20 ^c^
Monosaccharides composition (Molar ratio, %)			
Rha	11.92	6.82	4.77
Ara	34.87	44.45	67.42
Gal	17.74	32.51	6.88
Glc	5.72	4.04	5.90
Xyl	13.08	2.93	6.47
Man	5.79	2.48	3.23
GalA	9.56	5.84	4.60
GlcA	1.32	0.92	0.72
Molecular weight distribution (kDa)			
Peak 1	50.83	40.53	27.09
Content (%)	85.8	86.8	89.5
PDI ^C^ (M_w_/M_n_)	2.249	1.929	1.292
Peak 2	13.27	16.29	21.55
Content (%)	4.7	3.8	10.5
PDI ^C^ (M_w_/M_n_)	1.069	1.031	1.292
Peak 3	28.56	16.29	
Content (%)	9.5	9.3	
PDI ^C^ (M_w_/M_n_)	1.682	1.932	

Note: ^A^ The data were presented as “mean ± standard deviation (*n* = 3)”. ^B^ The data were presented as the degree of esterification. ^C^ PDI was presented as a polydispersity index. Values marked with different lowercase letters in the same row indicate significant differences (*p* < 0.05).

**Table 2:** Information on the range of particle size and height and the average roughness of the polysaccharide chains in the image of EP50, EP70, and EP90.

Images	EP50	EP70	EP90
Particle size range (nm)	31–65	31–114	31–55
Height range (nm)	2.541–9.478	1.531–7.657	1.642–7.222
Average roughness (nm)	0.869	0.435	0.217

**Table 3:** The fitting curve equation of double logarithmic plot of molecular weight against radius of gyration and the parameters “k” and “ν” of the power law functions (<S^2^>z^1/2^ = kM_w_ν) of EP50, EP70, and EP90.

Samples	Fitting Curve Equation	R^2^	k	ν
EP50	y = 0.2658x − 0.2173	0.9992	0.8047	0.2658
EP70	y = 0.2148x + 0.2783	0.9991	0.7571	0.2148
EP90	y = 0.2076x + 0.91	0.9997	0.4025	0.2076

Note: “y” was presented Ln <S^2^>z^1/2^; “x” was presented Ln M_W_.

## Data Availability

The original contributions presented in this study are included in the article/Supplementary Materials. Further inquiries can be directed to the corresponding authors.

## Supplementary Materials

The following supporting information can be downloaded at: https://www.mdpi.com/article/10.3390/foods14050791/s1, Supplementary Methods S1–S6. S1. The Detailed Extraction Process of the Determination of Esterification Degree; S2. Determination Method of Monosaccharide Composition; S3. Methods for Determination of Molecular Weight Distribution and Molecular Conformation; S4 Method for Determination of Chain Conformation (Congo Red Test); S5. Determination of Inhibition Rate on α-Glucosidase of EPs; S6. Study on the Antioxidant Activity of EP In Vitro.
