# Supplementary material for: Polysaccharides from Exocarpium Citri Grandis: Graded Ethanol Precipitation, Structural Characterization, Inhibition of α-Glucosidase Activity, Anti-Oxidation, and Anti-Glycation Potentials
Source: Foods. 2025 Feb 25;14(5):791. doi: 10.3390/foods14050791 (PMC11899376; doi:10.3390/foods14050791)
Supplement: Supplementary file 1 [file foods-14-00791-s001.zip › foods-3439019-supplementary.pdf]

Supplementary data for

Polysaccharides from *Exocarpium Citri Grandis*: Graded Ethanol Precipitation, Structural Characterization, Inhibition of α-Glucosidase Activity, Anti-Oxidation, and Anti-Glycation Potentials

Supplementary Methods

S1. The Detailed Extraction Process of the Determination of Esterification Degree

Infrared Spectrum Determination Method

The degree of esterification of EP was determined by the absorption peak area in the Fourier transform infrared spectroscopy. The esterification degree was calculated according to Formula (A.1).

$$\mathbf{DE = A_{1746}/(A_{1746} + A_{1627})\ \ (A.1)}$$

Where A_{1746} and A_{1630} represent the area of absorption peaks at 1746 cm^{-1} and 1627 cm^{-1}, respectively.

Chemical Titration

A total of 100 mg of EP was accurately weighed, and 100 mL of distilled water was added to fully dissolve it. After that, two drops of phenolphthalein reagent were added, and we recorded the titration volume V_1 when the solution was titrated with 0.1 mol/L NaOH to pink. Subsequently, 2.5 mL of 3.0 mol/L NaOH was added and stirred evenly. Then, 2.5 mL of 3.0 mol/L HCl was added and mixed well. The

solution was oscillated until it was no longer pink. Two drops of phenolphthalein reagent were added again, and the solution was titrated again with 0.1 mol/L of NaOH until the solution became pink again, and its color did not change in 1 min. At this time, the titration volume V_2 was recorded, and the degree of esterification was calculated by Equation (A.2).

$$\mathbf{DE = V_1/(V_1 + V_2)} \quad \mathbf{(A.2)}$$

Where V_1 is the initial titration of the sample solution, mL; V_2 is the saponification titration of the sample solution, mL; DE is esterification degree, %.

S2. Determination Method of Monosaccharide Composition

We used Thermo ICS5000 ion chromatography system (ICS5000, Thermo Fisher Scientific, USA) equipped with Dionex TM CarboPac TM PA20 (150 mm×3 mm, 10 μm) and pulsed amperometric detector. A total of 3 mg of EP was added to 1 mL of 2 mol/L of trifluoroacetic acid (TFA) and heated at 121°C for 2 h. Nitrogen and methanol were used to remove excess trifluoroacetic acid, and the hydrolysate was dissolved in distilled water, which was diluted to the appropriate concentration and transferred into a chromatographic bottle for testing.

S3. Methods for Determination of Molecular Weight Distribution and Molecular Conformation

High-Performance Size Exclusion Chromatography-Multi-Angle Laser Light Scattering-Refractive Index Detection (HPSEC-MALLS-RI) was used to determine the absolute molecular weight (M_w), polydispersity index (M_w/M_n), and radius of

gyration ($<S^2>_z^{1/2}$) of EP. A total of 4.0 mg of EP was dissolved in 0.2 mol/L of NaCl, prepared into a sample solution of 2.0 mg/mL, and passed through a 0.22 μm microporous membrane, after which we collected filtrates and quantitatively injected them into the inlet of the liquid chromatograph for measurement.

The SEC column (Shodex, Japan) was composed of OHpak SB-806 HQ (300×8 mm), OHpak SB-804 HQ (300 × 8 mm), and OHpak SB-8043 HQ (300 × 8 mm) in series. The mobile phase was 0.2 mol/L of NaCl. The flow rate was 1.0 mL/min. The detectors were a differential detector (RID-G7162A) and a DAWN detector (Wyatt Technology Co.). The dn/dc value of each component was 0.138 mL/g. The data obtained from the SEC-MALLS-RI system were calculated and analyzed using ASTRA 8 (Wyatt Technology, CA, USA) software.

S4 Method for Determination of Chain Conformation (Congo Red Test)

Table S1. Composition of Congo red experimental system.

Concentration of NaOH (mol/L)	Polysaccharide (mL)	Congo red (mL)
0	2	2
0.1	2	2
0.2	2	2
0.3	2	2
0.4	2	2
0.5	2	2

The EP solution (1 mg/mL) was mixed with Congo red solution (100 μmol/L) by

stirring, and then we added 1 mol/L of NaOH solution until the final concentration in the solution was 0, 0.10, 0.20, 0.30, 0.40, and 0.50 mol/L, respectively. A mixed alkaline solution without polysaccharides was used as a negative control, and curdlan was used as a positive control. After 10 min of the reaction, the maximum absorption wavelength (λ_{max}) in the range of 400 nm to 800 nm was recorded using an ultraviolet spectrophotometer at room temperature.

S5. Determination of Inhibition Rate on α-Glucosidase of EPs

Preparation of Experimental Solutions

(1) α-glucosidase masterbatch: A total of 5 mL of phosphate-buffered saline (PBS) was added to the reagent bottle containing α-glucosidase powder of 100 U to prepare the α-glucosidase masterbatch of 20 U/mL, of which 1.0 mL was taken and diluted to 0.5 U/mL in a centrifuge tube (50 mL). The remaining enzyme solution was frozen and stored at -20°C.

(2) 4-Nitrophenol-α-D-glucopyranoside (PNPG) solution: A total of 37.6563 mg of PNPG was dissolved in distilled water to configure it to 25 mL of a 5 mmol/L concentration.

(3) Na_2CO_3 solution: A total of 2.1198 g of Na_2CO_3 was weighed, and we added distilled water to prepare 100 mL of a 0.2 mol/L Na_2CO_3 solution.

(4) Different concentrations of EP and acarbose solution: The mass concentration gradients of EP and acarbose were 0.03125, 0.0625, 0.125, 0.25, 0.5, 1, 2, 4, and 8 mg/mL, respectively.

Study on the Inhibitory Effect on α-Glucosidase

A total of 40 μL of sample was added to 96-well plates, and then 40 μL of 0.5 U/mL glucosidase solution was added, mixed evenly, and incubated at 37 °C for 10 min. After adding 20 μL of 5 mmol/L of PNPG solution, it was incubated at 37 °C for 20 min, and finally, 100 μL of 0.2 mol/L of Na_2CO_3 solution was added to end the reaction. The absorbance was measured at 405 nm wavelength. With 0.1 mol/L PBS (pH 6.8) instead of the sample as a blank, the test group was divided into six groups, with three parallels in each group, respectively.

(1) Sample groups: EPs+α-glucosidase+PNPG

(2) Sample background groups: EPs+PBS+PNPG

(3) Positive control groups: Acarbose+α-glucosidase+PNPG

(4) Positive control background groups: Acarbose+PBS+PNPG

(5) Blank groups: PBS+α-glucosidase+PNPG

(6) Blank background groups: PBS+PNPG

Table S2. The measurement experiment of α-glucosidase inhibitory activity.

Groups	Polysaccharides or acarbose (μL)	α-glucosidase (μL)	PNPG (μL)	PBS (μL)	Na_2CO_3 (μL)
Polysaccharides or positive control group	40	40	20	0	100
Polysaccharides or positive control	40	0	20	40	100

background group					
Blank group	0	40	20	40	100
Blank background group	0	0	20	80	100

The inhibition rate of each sample on α-glucosidase was calculated according to Formula (A.3).

$$\textbf{Inhibition rate}\ (\%) = [\mathbf{1} - (\mathbf{E_1} - \mathbf{E_2})/(\mathbf{E_3} - \mathbf{E_4})] \times \mathbf{100} \quad \textbf{(A.3)}$$

In Formula (A.3),

E_1 is the absorbance of the sample groups/positive control groups;

E_2 is the absorbance of the sample/positive background groups;

E_3 is the absorbance of the blank group;

E_4 is the absorbance of the blank background groups.

S6. Study on the Antioxidant Activity of EP In Vitro

DPPH Free Radical Scavenging Activity

The mass concentration gradients of EP and ascorbic acid solution were 0.1, 0.2, 0.4, 0.8, 1.6, and 3.2 mg/mL, respectively. A total of 1 mL of polysaccharides with different concentrations was mixed with 1 mL of freshly prepared 0.1 mmol/L DPPH (dissolved in methanol), placed at room temperature in dark for 30 min, and then added to a 96-well plate to determine the absorbance of the solution at 517 nm. Ascorbic acid was used as a positive control group, and distilled water was used instead of the sample solution as a blank group. DPPH free radical scavenging rate

was calculated according to Formula (A.4).

$$\textbf{DPPH free radical scavenging rate}\ (\%) = [\mathbf{1} - (\mathbf{A_1} - \mathbf{A_2})/(\mathbf{A_0})] \times \mathbf{100} \quad \textbf{(A.4)}$$

In Formula (A.4),

A_0 was the absorbance of the blank group (distilled water instead of the sample solution); A_1 was the experimental group (EP/ascorbic acid) absorbance; A_2 was the absorbance of the background group (methanol instead of DPPH solution).

ABTS Free Radical Scavenging Activity

Distilled water was used to prepare 7.4 mM of ABTS solution and 2.6 mM of potassium persulfate solution, respectively. The ABTS solution and potassium persulfate solution were mixed at 1:1 (volume ratio) and stored at room temperature in dark for 24 h. After incubation, a small amount was diluted 45 times with distilled water to obtain an ABTS working solution. A total of 0.2 mL of diluted ABTS solution was mixed with 0.1 mL of different concentrations of EP solution, reacted at room temperature for 15 min, and added to a 96-well plate, and the absorbance was measured at 734 nm. Ascorbic acid was used as a positive control group, and distilled water was used instead of the polysaccharide sample as a blank group. The ABTS free radical scavenging ability was calculated according to Formula (A.5).

$$\textbf{ABTS free radical scavenging rate}\ (\%) = [\mathbf{1} - (\mathbf{A_a} - \mathbf{A_b})/(\mathbf{A_0})] \times \mathbf{100} \quad \textbf{(A.5)}$$

In Formula (A.5),

A_0 was the absorbance of the blank group (distilled water instead of sample solution);

A_1 was the absorbance of the experimental group (EP/ascorbic acid);

A_b was the absorbance of the background group (distilled water instead of ABTS solution).

Oxygen Radical Absorbance Capacity

Before the experiment, 0.5 mg/mL of EP solution (EP50, EP70 and EP90), water-soluble vitamin E (Trolox) standard solution (3.125, 6.25, 12.5, 25, 50, 100 μmol/L), 95.6 nmol/L of fluorescein (FL) working solution, and 200 mmol/L of AAPH solution were prepared. All solutions were dissolved in PBS (75 mmol/L, pH 7.4). A total of 25 μL of Trolox standard solution of different concentrations was added to the micropores of 96-well plates and incubated in a microplate reader at 37°C for 10 min. After that, 150 μL of FL working solution was added to each well, shocked and mixed, and 25 μL of AAPH solution was added quickly after the 96-well plates were taken out. After mixing, the measurement began immediately. The fluorescence values at the excitation wavelength of 485 nm and the emission wavelength of 538 nm were measured, and the fluorescence was recorded every 5 min for 125 min so that 25 sets of fluorescence values were recorded.

$$\mathbf{AUC = (0.5 + f_2/f_1 + f_i/f_1 + \cdots + f_{25}/f_1 + 0.5 \times f_{25}/f_1) \times 5} \quad \textbf{(A.6)}$$

Among them, fi was the fluorescence intensity measured for the ith time.

The net AUC value was obtained by subtracting the area under the fluorescence decay curve (AUC_{Trolox}) under the action of the Trolox standard from the area under the fluorescence decay curve (AUC_{blank}) of the blank group. As Formula (A.7) shows,

$$\textbf{net } \mathbf{AUC=AUC_{Trolox}-AUC_{blank}} \quad \textbf{(A.7)}$$

The standard curve of net AUC was drawn with the concentration of Trolox

standard, and the fitting formula was obtained by linear fitting. Thus, the Trolox equivalent (μmol Trolox/g DW) of the antioxidant capacity of EP can be calculated.

S6. Study on the Inhibitory Effect of Polysaccharides from ECG on the Products of Non-Enzymatic Glycation

Establishment of Non-Enzymatic Glycation System of Bovine Serum Albumin-Glucose In Vitro

Table S3. Composition of the system of BSA-Glucose model.

Groups	Test no.	BSA (mL)	Glu (mL)	EP/AG (mL)	PBS (mL)	Total (mL)
Experimental group	1	1.5	1.5	0.05	1.95	5
	2	1.5	1.5	0.25	1.75	5
	3	1.5	1.5	0.5	1.5	5
	4	1.5	1.5	1	1	5
Experimental background group	5	1.5	0	0.05	3.45	5
	6	1.5	0	0.25	3.25	5
	7	1.5	0	0.5	3	5
	8	1.5	0	1	2.5	5
Blank group	9	1.5	1.5	0	2	5
Blank background group	10	1.5	0	0	3.5	5

The bovine serum albumin (BSA)-Glucose (Glu) reaction system was used as the saccharification model, which was established under sterile conditions. The EP sample and aminoguanidine (AG) were prepared into 10 mg/mL of solution, respectively, and 20 mg/mL of BSA solution and 0.50 mol/L of glucose solution were prepared. The four solutions needed to be sterilized using a 0.22 μm microporous membrane before being used. After sterilization, 1.5 mL of BSA solution, 1.5 mL of glucose solution, a certain amount of EP/AG solution, and 0.20 mol/L of PBS (pH 7.4) were added to a 5 mL centrifuge tube, so that the final concentrations of EP or AG in the solution were 0.1 mg/mL, 0.5 mg/mL, 1.0 mg/mL, and 2.0 mg/mL, respectively. AG was used as a positive control, and PBS was used as a blank control. The mixtures were cultured at 37°C in an incubator for 15 days and were taken on the 1st, 5th, 10th, and 15th days for measurement, respectively. As shown in Table S3, the experimental groups were 1, 2, 3, and 4, and the experimental background groups were 5, 6, 7, and 8. The blank group was 9, and the blank background group was 10, with three parallels in each group.

(1) Experimental groups:

EP+BSA+Glu+PBS (Samples); AG+BSA+Glu+PBS (Positive control)

(2) Experimental background groups:

EP+BSA+PBS (Samples background); AG+BSA+PBS (Positive control background)

(3) Blank groups: BSA+Glu+PBS

(4) Blank background groups: BSA+PBS

Determination of Inhibition Rate of the First Stage Product (Amadori Product)

of Glycosylation

The Amadori products in the first stage were determined by a nitro blue tetrazolium chloride (NBT) reduction experiment. The specific experimental operations were as follows:

On the 1st, 5th, 10th, and 15th day of culture, 0.5 mL of a saccharification sample solution of each group was added to a 5 mL centrifuge tube, and then 2.0 mL of 0.3 mmol/L NBT solution was added. Finally, 2.5 mL of 100 mmol/L sodium carbonate buffer solution (pH=10.35) was added and incubated at room temperature (37°C) for 1 h. The absorbance was measured at a wavelength of 530 nm. The sodium carbonate buffer solution was used instead of the saccharification solution as a blank group. The inhibition rate of the Amadori product was calculated according to Formula (A.8).

$$\textbf{Inhibition rate}\ (\%) = [\mathbf{1} - (\mathbf{A_1} - \mathbf{A_2})/(\mathbf{A_3} - \mathbf{A_4})] \times \mathbf{100} \quad \textbf{(A.8)}$$

In Formula (A.8),

A_1—The absorbance of the experimental group;

A_2—The absorbance of the experimental background group;

A_3—The absorbance of the blank group;

A_4—The absorbance of the blank background group.

Determination of the Content of the Product (Dicarbonyl Compound) in the Second Stage of Glycosylation

The standard curve of glyoxal, such as in Fig.1, was drawn to calculate the content of dicarbonyl compounds in the second stage of glycosylation. The specific

experimental operations are as follows:

Preparation of standard curve: The glyoxal standard solution (concentration of 0, 0.02, 0.04, 0.08, 0.16, and 0.32 mmol/L) was prepared, and 0.4 mL of each solution was mixed with 0.2 mL of 500 mmol/L Gillard-T stock solution and 3.4 mL of sodium formate solution (pH=2.9) and incubated at room temperature (37°C) for 1 h. The sodium formate solution was used to replace the saccharification solution as a blank group, and the absorbance was measured at 294 nm. The standard curve was drawn with the concentration of glyoxal as the abscissa and the absorbance as the ordinate.

Determination of saccharification products: On the 1st, 5th, 10th, and 15th days of culture, the saccharification substances of the 2 mg/mL polysaccharide group were taken instead of the glyoxal standard, and the operation was performed according to the above standard curve operation method. Each group was tested three times in parallel, and the corresponding dicarbonyl compound content was calculated by substituting the standard curve.

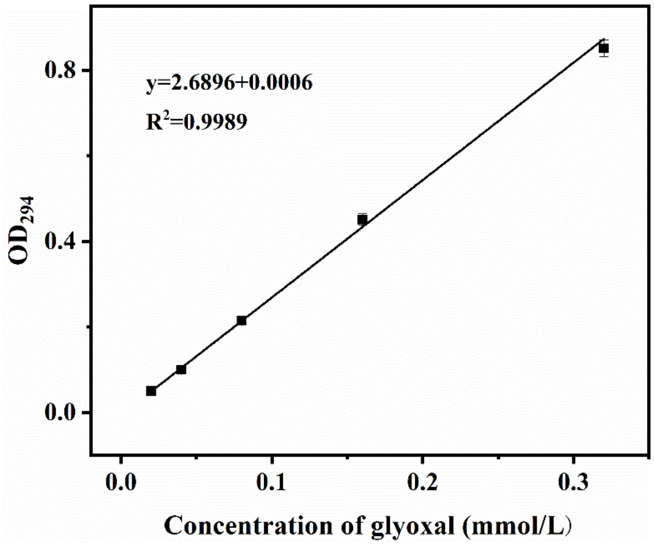

Fig. S1 The standard curve of glyoxal

Determination of Inhibition Rate of Non-Enzymatic Glycation End Products (AGEs)

A total of 0.5 mL of saccharification solution in each concentration group was diluted to 10 mL with PBS (200 mmol/L, pH=7.4) on the 15th day of the reaction. The fluorescence value was measured at the excitation wavelength (Ex) 370 nm and at the emission wavelength (Em) 440 nm, and the inhibition rate was calculated according to Formula (A.5).

$$\textbf{Inhibition rate}\ (\%) = [\mathbf{1} - (\mathbf{F_1} - \mathbf{F_2})/(\mathbf{F_3} - \mathbf{F_4})] \times \mathbf{100} \quad \textbf{(A.9)}$$

In Formula (A.9),

F_1—The fluorescence values of the experimental group;

F_2—The fluorescence values of the experimental background group;

F_3—The fluorescence values of the blank group;

F_4—The fluorescence values of the blank background group.
